# No evident association between efavirenz use and suicidality was identified from a disproportionality analysis using the FAERS database

**DOI:** 10.7448/IAS.17.1.19214

**Published:** 2014-09-04

**Authors:** Andrew A Napoli, Jennifer J Wood, John J Coumbis, Amit M Soitkar, Daniel W Seekins, Hugh H Tilson

**Affiliations:** 1 Bristol-Myers Squibb, Virology, Plainsboro, NJ, USA; 2 Bristol-Myers Squibb, Global Pharmacovigilance and Epidemiology, Hopewell, NJ, USA; 3 UNC Gillings School of Global Public Health, Public Health Leadership Program, Chapel Hill, NC, USA

**Keywords:** HIV, efavirenz, antiretroviral, suicidality, disproportionality, Food and Drug Administration Adverse Event Reporting System, Multi-Item Gamma Poisson Shrinker

## Abstract

**Objective:**

To assess the potential association of selected antiretrovirals (ARVs), including efavirenz, with suicidality.

**Design:**

Retrospective analysis of the Food and Drug Administration Adverse Event Reporting System (FAERS), by performing a Multi-Item Gamma Poisson Shrinker (MGPS) disproportionality analysis.

**Methods:**

MGPS disproportionality analysis, a technique to identify associations between drugs and adverse events, was performed using cumulative data from the FAERS database collected up to August 2012. This method yields an Empirical Bayesian Geometric Mean score and corresponding 90% confidence interval (EB05, EB95). EB05 scores ≥2 were pre-defined as a signal for a potential drug-event association. The FAERS database includes spontaneous adverse-event reports from consumers and healthcare professionals. All FAERS reports of suicidality (including suicidal ideation, suicide attempt and completed suicide or a composite of these) in patients taking efavirenz (as single agent or in fixed-dose combination), atazanavir, darunavir, etravirine, nevirapine and raltegravir were identified. A number of parallel analyses were performed to assess the validity of the methodology: fluoxetine and sertraline, antidepressants with a known association with suicidality, and raltegravir, an ARV with rhabdomyolysis and myopathy listed as “uncommon” events in the US-prescribing information.

**Results:**

A total of 29,856 adverse event reports were identified among patients receiving efavirenz, atazanavir, darunavir, etravirine, nevirapine and raltegravir, of which 457 were reports of suicidality events. EB05 scores observed for the composite suicidality term for efavirenz (EB05=0.796), and other ARVs (EB05=0.279–0.368), were below the pre-defined threshold. Fluoxetine and sertraline gave EB05 scores for suicidality >2. Raltegravir gave EB05 scores >2 for myopathy and rhabdomyolysis.

**Conclusions:**

The pre-determined threshold for signals for suicidality, including suicidal ideation, suicide attempt, completed suicide and a composite suicidality endpoint, was not exceeded for efavirenz and other ARVs in this analysis. Efavirenz has been associated with suicidality in clinical trials. Further studies that adjust for confounding factors are needed to better understand any potential association with ARVs and suicidality.

## Introduction

Efavirenz is a non-nucleoside reverse transcriptase inhibitor for the treatment of HIV infection in combination with other antiretrovirals (ARVs). Rare but serious psychiatric adverse events, including severe depression, suicidal ideation, non-fatal suicide attempts and completed suicide, have been reported in clinical trials and in post-marketing surveillance among patients taking efavirenz [1–4]. Recently, a pooled analysis of four AIDS Clinical Trial Group (ACTG) studies, which included 5332 subjects, identified an increased risk of suicidality in efavirenz-containing regimens compared to regimens containing atazanavir, atazanavir/ritonavir, lopinavir/ritonavir and zidovudine/lamivudine/abacavir [5].

The Food and Drug Administration Adverse Event Reporting System (FAERS, formerly AERS) is a database set up to support the FDA's post-marketing surveillance programme by recording adverse events spontaneously reported by consumers and healthcare professionals to the FDA or manufacturers [6]. As there is no measure of the total number of patients exposed to a particular drug in a spontaneously reported adverse event database, it is not possible to estimate the rate of adverse events. Drug-event pair disproportionality analysis is a method to identify potential signals for drug-associated adverse events using spontaneous adverse event reporting surveillance databases [7–9]. Disproportionality analysis compares the frequency that a selected adverse event is reported with a given drug, to the frequency it is reported with other drugs. Simple methods for disproportionality analysis can be utilized, but when the number of events is small, large estimates with wide confidence intervals can lead to many false-positive reporting rates for very rare events [10, 11]. A well-established technique to minimize false-positive reporting rates is Multi-Item Gamma Poisson Shrinker (MGPS) disproportionality analysis, which applies a Bayesian shrinkage estimator to the relative reporting ratio (RRR; the ratio of the observed frequency to the expected frequency), to give smaller risk estimates with narrower confidence intervals, even when event counts are small [10, 11].

The aim of this analysis was to assess the potential association of selected ARVs, including efavirenz, with suicidality by performing an MGPS disproportionality analysis of the FAERS database.

## Methods

### Search of FAERS database

The analysis was performed using all cumulative data available from the FAERS database collected from 1968 up to 27 August 2012. The drugs of interest for the analysis, selected for common use and mechanism of action, were efavirenz and other ARVs, atazanavir, darunavir, etravirine, nevirapine and raltegravir. For efavirenz, the records identified included those in which efavirenz was administered alone or as part of the fixed-dose combination product containing efavirenz, emtricitabine and tenofovir disoproxil fumarate.

The Medical Dictionary for Regulatory Activities (MedDRA version 15.0) preferred terms selected for identification of adverse events were “suicidal ideation,” “suicide attempt” and “completed suicide” to match as closely as possible the methods used in the ACTG-pooled analysis [5]. An additional analysis was performed using a composite suicidality endpoint in which all reports associated with “suicidal ideation,” “suicide attempt” or “completed suicide” were combined. The individual term analysis provides the total number of suicidality events reported, whereas the composite term generates unique “suicidality” reports per drug, as two events of interest could be identified in the same report. A number of parallel analyses were performed to assess the validity of the methodology: fluoxetine and sertraline, antidepressants with a known association with suicidality [12], and a sensitivity analysis with raltegravir, an ARV with rhabdomyolysis and myopathy listed as “uncommon” (defined as between 1% and 0.1%) events in the US-prescribing information [13–15].

Duplicate records were deleted using a removal algorithm on retrieved reports. Because adverse event reports are collected from different sources such as consumers, drug manufacturers and investigators, multiple reports referring to the same adverse events are not uncommon in FAERS [10]. In order to unmask the effects of duplicate reports on signal scores, the proprietary database software Oracle performs automated duplicate detection on FAERS data. The duplicate detection process is organized in multiple stages, each stage defining “duplicate groups” based on matching records. Matching is performed by considering equivalence in demographic fields (same gender, same or nearby age values, death date if present) and a combination of manufacturer, drug and event information, including drug and/or event start dates. The algorithm also considers the report source (i.e. spontaneous versus literature or study) and adjusts the criteria accordingly.

### Statistical analyses

The measure of disproportionality was defined in terms of the RRR of observed to expected frequencies of reports mentioning both the selected drug (Y) and the selected adverse event (X), where:

Observed count = Number of reports for event X with drug Y,

$$\begin{array}{c} \text{Expected count}=\frac{\left(\text{Number of reports for event X})*(\text{Number of reports for drug Y}\right)}{\text{Total number of reports}}\text{and}\\ \text{RRR}=\frac{\text{Observed count}}{\text{Expected count}} \end{array}$$

The expected frequency of a drug-event is typically calculated under the assumption of independence between drug and event distributions. Therefore, this measure of disproportionality is viewed also as a measure of association. Disproportionality analysis was performed using the MGPS method. MGPS is a Bayesian method that provides a robust estimate of the disproportionality measure, RRR, which is referred to as the Empirical Bayesian Geometric Mean (EBGM) and its corresponding 90% confidence interval (EB05, EB95). EBGM is an estimate of the ratio of observed to expected number of drug-event reports. EB05 values indicate about a 5% probability that the true value of RRR (i.e. observed/expected) falls below it. EB95 values indicate about a 5% probability that the true value of RRR (i.e. observed/expected) falls above it. EB05 scores ≥2 (at least two times greater than expected) indicate a potential signal for a drug-event association [16, 17]. This threshold identifies drug-event combinations that are reported at least twice as often as all other drug-event combinations and was selected to provide sensitivity for the detection of potential rare drug-event combinations [16, 17].

## Results

The FAERS database included 29,856 adverse event reports among patients receiving efavirenz, atazanavir, darunavir, etravirine, nevirapine and raltegravir, including 197 reports of suicidal ideation, 182 suicide attempts and 78 completed suicides (457 events in total).

**Figure 1:**
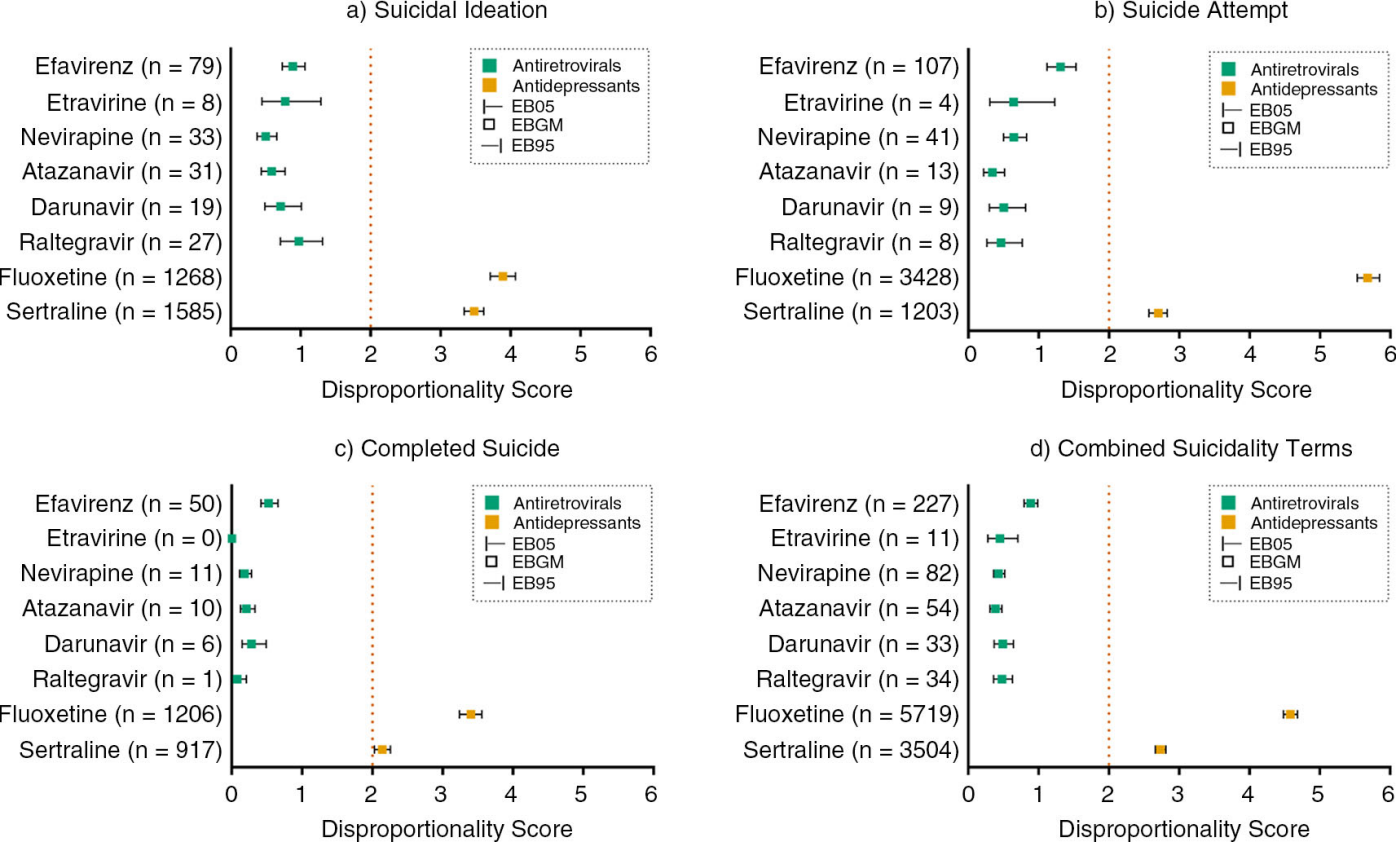
MGPS signal scores of drug-event pairs for a) suicide ideation, b) suicide attempt, c) completed suicide and d) combined suicidality terms. EBGM, Empirical Bayesian Geometric Mean; EB05, lower limit of the EBGM 90% confidence interval; EB95, EBGM upper limit of the EBGM 90% confidence interval; *n*=number of reports.

Measures of disproportionality (EBGM, EB05 and EB95) for efavirenz, other ARVs, fluoxetine and sertraline are provided in Figure 1. Disproportionality scores (EB05) for suicidality observed for efavirenz were below the threshold of 2. Efavirenz EB05 scores for suicidal ideation, suicide attempt and completed suicide were 0.737, 1.118 and 0.416, respectively. Likewise, all other ARVs did not exceed the threshold EB05 score for suicidal ideation (0.377–0.711), suicide attempt (0.218–0.501) or completed suicide (0.023–0.149). Analysis of the composite suicidality term for efavirenz produced an EB05 score of 0.796, which was also below the threshold, as were scores for all other ARVs (0.279–0.368).

Fluoxetine and sertraline had EB05 scores ≥2 for suicidal ideation, suicide attempt and completed suicide (Figure 1). Analysis of the composite suicidality term for fluoxetine and sertraline produced EB05 scores of 4.48 and 2.66, respectively (Figure 1). In the sensitivity analysis for myopathy and rhabdomyolysis, raltegravir gave EB05 scores of 2.334 and 2.333, respectively.

## Conclusions

In this analysis, the level of disproportionality did not exceed the pre-determined threshold of two for suicidality among patients receiving efavirenz, or other ARVs. A potential signal for suicidality was identified for the two SSRIs. Furthermore, our analysis was sensitive enough to detect a potential signal for uncommon adverse events with raltegravir. This is the first analysis performed using a real-world dataset to assess the association of efavirenz, or other ARVs, with suicidality.

Serious psychiatric adverse experiences have been reported in patients treated with efavirenz in clinical trials [1, 5]. In controlled trials, the frequency (regardless of causality) of specific serious psychiatric events among patients who received efavirenz versus control regimens were severe depression (2.4% vs. 0.9%), suicidal ideation (0.7% vs. 0.3%), non-fatal suicide attempts (0.5% vs. 0), aggressive behaviour (0.4% vs. 0.5%), paranoid reactions (0.4% vs. 0.3%) and manic reactions (0.2% vs. 0.3%) [1]. While an association of psychiatric adverse events with EFV has been reported, the relative magnitude of this risk compared to other ARVs is less clear. The recently reported pooled analysis of four ACTG studies reported a two-fold increase in risk of suicidality with efavirenz-containing regimens [5]. However, the ACTG analysis was retrospective using pooled data from four studies, three of which were open label and none of which included recognized psychiatric measures of suicidality or depression [5]; limitations that also apply to the current FAERS analysis.

Patients tend to underreport psychiatric illness [18, 19], and healthcare providers do not systematically screen patients for symptoms of psychiatric illnesses, which will impact assessments in both clinical trials as well as observational approaches [20, 21]. Furthermore, warnings of suicide risk have been reported in the efavirenz US package insert since 1998 [1], which may have led to ascertainment bias and/or over- or underreporting in our FAERS analysis. Patients may seek treatment for low mood or suicide ideation with non-HIV physicians (who may be less aware of the label warning), thus potentially further contributing to underreporting. Channelling bias is also likely since providers who are aware of the label warning would be expected to avoid prescription of efavirenz in patients with a known history of mental health problems.

The FAERS database was selected for this analysis based on it being a large publicly accessible database from a heterogeneous patient population. However, there are a number of established limitations to these data. For example, because the database primarily includes spontaneous or voluntary reports of adverse events, these records can be of varying quality and it is known that events are underreported [10]. Reporting patterns may be influenced by marketing and publicity about specific adverse events; the so-called “notoriety effect” [22, 10]. The number of treated patients is not known, so reporting rates cannot be calculated. It should also be noted that while disproportionality analysis can identify signals for potential correlations between drugs and adverse events, it can neither determine whether there is a direct cause-and-effect relationship nor quantify risk. The failure of efavirenz to exceed the pre-determined threshold for suicidality in this analysis must not be misinterpreted as an absence of association, given the limitations of these methods.

The results presented here were derived from spontaneous reports based on actual clinical experience and hence they complement those derived from randomized controlled clinical trials. Clinical trials have previously identified an increased risk of depression and suicidality with efavirenz treatment [1, 5]. The association between efavirenz use and suicidality, as reflected in the product labelling warning, should be kept in mind when making treatment decisions. The risk of depression and suicidality among persons diagnosed with HIV infection indicates that, irrespective of ARV therapy choice, psychiatric screening and counselling are important aspects of clinical management [23]. Future studies of experiences in randomized clinical trials or in routine clinical practice would further elucidate any potential associations between efavirenz use and suicidality.
